# Hydrothermally treated coral scaffold promotes proliferation of mesenchymal stem cells and enhances segmental bone defect healing

**DOI:** 10.3389/fbioe.2023.1332138

**Published:** 2023-12-20

**Authors:** Jianping Huang, Jaehan Park, Narae Jung, Hong Seok Moon, Zhixian Zong, Gang Li, Sien Lin, Sung-Won Cho, Youngbum Park

**Affiliations:** ^1^ Department of Prosthodontics, College of Dentistry, Yonsei University, Seoul, Republic of Korea; ^2^ Musculoskeletal Research Laboratory, Department of Orthopaedics and Traumatology, The Chinese University of Hong Kong, Prince of Wales Hospital, Hong Kong, China; ^3^ Stem Cells and Regenerative Medicine Laboratory, Li Ka Shing Institute of Health Sciences, The Chinese University of Hong Kong, Prince of Wales Hospital, Hong Kong, China; ^4^ Division of Anatomy and Developmental Biology, Department of Oral Biology, College of Dentistry, Yonsei University, Seoul, Republic of Korea

**Keywords:** bone defect, coralline materials, hydrothermal modification, mesenchymal stem cells, hydroxyapatite

## Abstract

**Introduction:** Synthetic hydroxyapatite (HAp) scaffolds have shown promising therapeutic outcomes in both animals and patients. In this study, we aim to evaluate the chemical and physical phenotype, biocompatibility, and bone repair effects of hydrothermally treated coral with natural coral and synthetic HAp.

**Methods:** The phase composition, surface pattern, 3D structures, and porosity of the scaffolds were characterized, and cell viability, proliferation, and osteogenic differentiation of mesenchymal stem cells (MSCs) after seeding onto the scaffold were determined. The scaffolds were implanted into rats to assess their bone repair effects using micro-CT analysis, mechanical testing, and histological staining.

**Results:** The results showed that the phase composition, porous structure, and porosity of hydrothermally treated coral were comparable to pure HAp scaffold. While only the natural coral happens to be dominantly calcium carbonate. Higher cell proliferation and osteogenic differentiation potential were observed in the hydrothermally treated coral scaffold compared to natural coral and pure HAp. Histological results also showed increased new bone formation in the hydrothermally treated coral group.

**Discussion:** Overall, our study suggests that hydrothermal modification enhances the cytocompatibility and therapeutic capacity of coral without altering its physical properties, showing superior effectiveness in bone repair to synthetic HAp.

## 1 Introduction

The importance of maintaining the normal structure and function of dental tissues for overall health has made dental tissue regeneration critical area of focus in modern dentistry (Nauta et al., 2011; Zhai et al., 2019). Among the dental tissues, bone has a very strong repair ability under normal circumstances, even in the case of fractures or other injuries, bone tissue can still be fully repaired (Battafarano et al., 2021). However, large bone defects exceeding the critical size caused by bone tumors, injuries, and other bone diseases typically require effective repair with the help of bone grafts to restore function (Schemitsch, 2017; Huang et al., 2022). Therefore, bone grafts play a very important role in the treatment of bone defects (Fernandez de Grado et al., 2018; Zhou et al., 2021). However, the availability of autologous bone is limited and may result in delayed union or non-union of the injured site (Wang and Yeung, 2017; Schmidt, 2021). Therefore, the research and development of biocompatible materials with osteoconductive properties for the treatment of bone defects are of great clinical significance.

Coral is a natural marine organism that is rich in calcium carbonate, which gives it its unique bone-like porous structure. Coral is also known for its high tissue compatibility and ability to promote bone repair. The use of coral as a bone substitute material has been studied for decades, and it has been found to be effective in the repair of bone defects in both animals and humans (Dorozhkin, 2010; Liu et al., 2013; Pountos and Giannoudis, 2016). However, the use of natural coral has some limitations, such as the variability in composition and impurities among different species and the limited availability of coral resources. What’s more, the differences in composition between coral and bone can affect the final bone defect repair effect (Pountos and Giannoudis, 2016). Coraline calcium carbonate can be absorbed very quickly after implantation *in vivo*, making it difficult for new bone tissue to grow onto the scaffold (Macha and Ben-Nissan, 2018). Therefore, attempts have been tried by converting coral into other forms of materials similarly to bone and maintaining the porous structure (Nandi et al., 2015; Balu et al., 2021). Those previous studies have suggested that converting coralline material from carbonate to phosphate could significantly delay its degradation, making it a promising material for bone grafting. However, no comprehensive study has been conducted to compare coral converted hydroxyapatite (HAp) with synthetic HAp in their compositions, morphology, mechanical properties, cellular responses, and efficacy in animal model. We thereby hypothesize that coral converted HAp may present a better bone healing outcome comparing with synthetic HAp and nature coral.

Herein, we prepared a coral converted HAp by treating natural coral with high temperature and hydrothermal treatment. In this study, we aim to evaluate the chemical and physical phenotype, biocompatibility, and bone repair effects of coral converted HAp with natural coral and synthetic HAp.

## 2 Materials and methods

### 2.1 Preparation of coral materials

The wild coral reefs were donated to Dr. David Green at the Faculty of Dentistry of the University of Hong Kong who collected from the Northeast Australia. The natural samples were first tailored into uniformly sized cylindrical structures with diameter 4 mm and height 2.5 mm. They were then washed three times with distilled water under ultrasound for 15 min each time to remove the salt in the coral. Afterwards, they were dried in a 60°C oven and washed again with acetone under ultrasound for 15 min. They were then dried overnight in a 70°C oven to obtain the natural coral (N-Coral) group samples.

Half number of the clean natural coral were placed in a 900°C oven for 2 h to remove the organic components, and to convert them into phosphate components accordingly to a previous method (Nandi et al., 2015). Then the treated scaffolds were soaked in 0.6 M di-ammonium hydrogen orthophosphate ((NH_4_)_2_HPO_4_, VWR Lab, India) solution and potassium di-hydrogen phosphate (KH_2_PO_4_, VWR Lab, India) solution and autoclaved at 150°C, followed by sintered at 1,250°C. (NH_4_)_2_HPO_4_ was used as source of phosphate group and KH_2_PO_4_ was used as a mineralizer. The proposed exchange reaction is shown below:

$$10{\text{CaCO}}_{3}+6\left({\text{NH}}_{4}\right){\text{HPO}}_{4}+2H_{2}O\to {\text{Ca}}_{10}{\left({\text{PO}}_{4}\right)}_{6}{\left(\text{OH}\right)}_{2}+6{\left({\text{NH}}_{4}\right)}_{2}{\text{CO}}_{3}+4H_{2}{\text{CO}}_{3}$$

Finally, the hydrothermally treated coral group (T-Coral) samples were obtained. An equal volume of hydroxyapatite (HAp, PENTAX Co., Japan) porous material with similar porosity was used as the control group samples.

### 2.2 Characterization of coral materials

Fourier transformed infra-red spectroscopy (FTIR) (Perkin-Elmer, United States) were adopted to analyze phase constituents of the N-Coral, T-Coral, and HAp. Field-emission Scanning electron microscopy (SEM; ZEISS SUPRA 40 VP, Germany) were used to determine the surface of porous cylindrical scaffolds (*n* = 3) at 3 kV under various magnifications. Micro-computed tomography (micro-CT) were used to measure the three-dimensional (3D) microstructure of the scaffolds (*n* = 3) at 70 keV, 114 mA, and isotropic resolution of 10.5 μm (μCT-40, Scanco Medical, Bassersdorf, Switzerland). The 3D reconstruction was performed with a segmentation parameter (sigma: 1.2, support: 1, threshold: 30 mg/cm^2^) (Lin et al., 2019). A specific gravity bottle (Hubbard, Hanil, Korea) was used to determine the porosity of the fabricated scaffolds through the ethanol immersion method as described previously (Lin et al., 2019).

### 2.3 Cell proliferation and differentiation assays

The porous cylindrical scaffolds (diameter 4 mm and height 2.5 mm, *n* = 4) were plated in a 48-well plate. Bone marrow derived mesenchymal stem cells (MSCs) expressing green fluorescence protein (GFP) were isolated from GFP transgenic rats (Genome Information Research Center, Osaka University). The MSCs were seeded in a density of 2 × 10^4^ cell per well. Fluorescent microscope or SEM was used to observe the cells growing on the surface of scaffolds. Cell proliferation was determined by Alarma Blue assay on day 0, 5, and 10, respectively. The MSCs were plated at 1 × 10^5^ cells/per scaffold in a 24-well plate and cultured in the basal medium until the cells reached confluence. The cells were then incubated in osteogenic induction medium, which is basal medium supplemented with 1 nM dexamethasone, 50 μM ascorbic acid, and 20 mM β-glycerolphosphate (all from Sigma-Aldrich). At day 0, 3 or 7, the cells were harvested and homogenized for RNA extraction with the RNeasy mini kit (Qiagen, Hilden, Germany). Complementary DNA quantification was performed by qRT-PCR using Step-One-Plus Real Time PCR Systems (Applied Biosystems, Carlsbad, CA). Relative gene expression was calculated with the 2^−△△CT^ formula. Primer sequences (5′ to 3′) were determined through established GenBank sequences (Forward primer of *Runx2*: CCGATGGGACCGTGGTT; reverse primer of *Runx2*: CAG​CAG​AGG​CAT​TTC​GTA​GCT. Forward primer of *Ocn*: GAG​CTG​CCC​TGC​ACT​GGG​TG; reverse primer of *Ocn*: TGG​CCC​CAG​ACC​TCT​TCC​CG. Forward primer of *Opn*: TCC​AAG​GAG​TAT​AAG​CAG​CGG​GCC​A; reverse primer of *Opn*: CTC​TTA​GGG​TCT​AGG​ACT​AGC​TTT). Forward primer of *β-actin*: CGT​AAA​GAC​CTC​TAT​GCC​AAC​AT; reverse primer of *β-actin*: CGG​ACT​CAT​CGT​ACT​CCT​GCT.

### 2.4 *In vivo* implantation in bone defects

**FIGURE 1:**
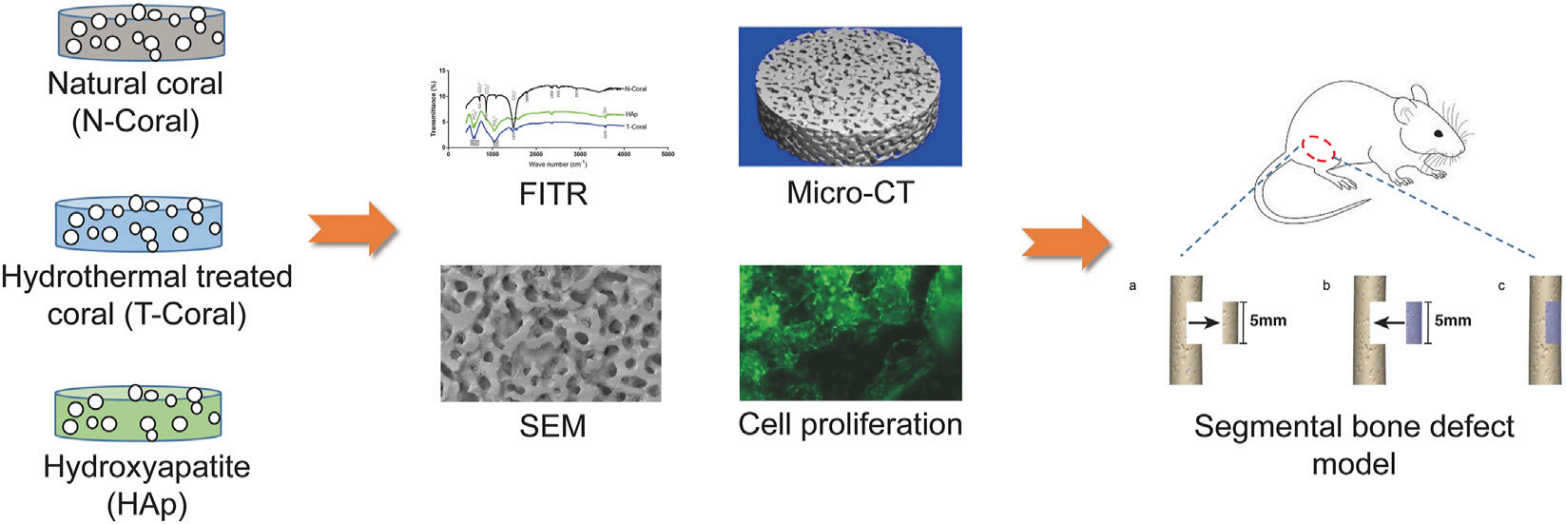
Schematic illustration of the study design. A coral-based graft was prepared by hydrothermal treatment (T-Coral). After conversion, the chemical and physical properties including the phase composition, surface morphology and porosity were analysed by FITR, SEM, and micro-CT, and compared with natural coral (N-Coral) and commercialized hydroxyapatite (HAp) product. Biological properties were further determined by cellular compatibility and bone defect healing after implantation in a segmental bone defect animal model.

Adult male SD rats (12-week-old, mean body weight of 350 g) were provided by the Laboratory Animal Research Centre of the Chinese University of Hong Kong with ethical approval from the Animal Experimentation Ethics Committee. For surgery, a solution of xylazine (2.5 mg/kg) and ketamine (50 mg/kg) was used for inducing general anaesthesia by intraperitoneal injection. Osteotomy was conducted to the mid-shaft to create a half-cortical bone defect, by removing a half cylinder with a diameter of 4 mm and a length of 5 mm (Figure 1). Animals were randomly selected for implantation of materials tailored into the same size of bone defects, including the raw coral control (*n* = 20), thermal treated coral material (*n* = 20), or HAp (*n* = 20). The bone was further secured with PEEK internal fixator before wound closure. Animals were euthanasia by inhalation of carbon dioxane for 10 min after 8 weeks of operations for sample harvesting.

### 2.5 Imaging assessment of affected femurs

The microstructure of the affected femurs (*n* = 10) was determined by Micro-computed tomography (micro-CT) analysis. The scanning parameters were 90 keV, 120 mA, and isotropic resolution of 10.5 μm using μCT-40 system (Scanco Medical, Bassersdorf, Switzerland). A segmentation parameter (sigma: 0.8, support: 2, threshold: 158–1,000 mg/cm^2^) for 3D reconstruction of images was applied. The volume of interest (VOI) was the defect area in femoral mid-shaft in a half-cylinder shape, with 4 mm in diameter. A total of 150 slices were used for quantification. Bone volume/tissue volume (BV/TV) was calculated with a built-in program in the μCT-40 system (Image Processing Language v4.29d, Scanco Medical, Switzerland).

### 2.6 Four-point bending mechanical test

Four-point bending test was conducted after micro-CT examination, the mechanical properties of specimens were evaluated accordingly to the methods reported before (Yang et al., 2020). After removal of PEEK fixator, a material testing system (H25KS; Hounsfield Test Equipment Ltd., United Kingdom) was adopted for measurement of the femoral load-to-failure with 2.5 kN load cell. The femurs were loaded on the support blades with inner and outer span set as 2 cm and 6 cm. A displacement rate of 5 mm/min on the mid-shaft of the femur was adopted during the compression. A built-in software (QMAT Professional; Tinius Olsen, Inc., Horsham, PA) was used to record the load versus displacement curves, and to calculate the maximum load, stiffness, and energy to failure.

### 2.7 Histological analysis

All the affected femurs (*n* = 10) were fixed with 4% PFA and then decalcified in 10% ethylenediaminetetraacetic acid (EDTA) solution. The decalcified femurs were subjected to paraffin embedding and then cut into 5 μm sections by a microtome (HM 355S; Thermo Fisher Scientific, MA). Hematoxylin (H) & Eosin (E) staining was conducted to identify different tissues inside the specimens, which further semi-quantitatively measured by ImageJ (NIH, United States). Expression of osteogenic marker, osteocalcin (OCN), was determined by immunohistochemistry staining with primary antibodies to OCN (1:200, Santa Cruz, TX) followed by counterstaining with hematoxylin. The positive stained area in the specimen were measured by ImageJ.

### 2.8 Statistical analysis

Sample size in animal study was determined by *a priori* power analysis (G*Power, Universität Düsseldorf). All the quantitative data were presented as mean and mean ± standard error of the mean (SEM), and the level of significance was set at *p* < 0.05. For mechanical testing, contralateral femurs were used to normalize the parameters. Data were analysed using GraphPad PRISM® (GraphPad Software, CA). One-way ANOVA was used for comparisons and Tukey’s HSD was used for the *post hoc* test. Statistical analysis was performed with SPSS (Version 20).

## 3 Results

### 3.1 Phase constituents

**FIGURE 2:**
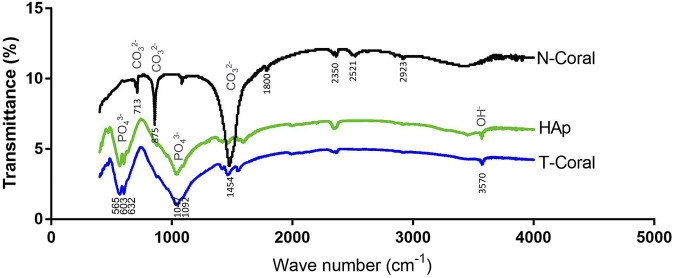
Fourier transformed infra-red spectroscopy (FTIR) spectra showing signature waveforms of CO_2_^−3^, PO_4_^3−^, and OH^−^ of the porous scaffolds, including nature coral (N-Coral), hydrothermally treated coral (T-Coral), hydroxyapatite (HAp).

From the results of FTIR (Figure 2), internal mode of CO_2_^−3^ (713, 875, 1,454 cm^−1^) were identified in the N-Coral samples. However, after hydrothermal conversion treatment, the presence of PO_4_^3−^ groups at 603 and 565 cm^−1^ (bending mode) and 1,047 cm^−1^ (stretching mode), and the presence of OH^−^ group of HAp were also observed at 3,570 cm^−1^, which patterns are nearly the same as HAp. A tiny peak of CO_3_ groups of the T-Coral at 1,454 cm^−1^ indicates the presence of residual carbonates.

### 3.2 Morphology and porosity

**FIGURE 3:**
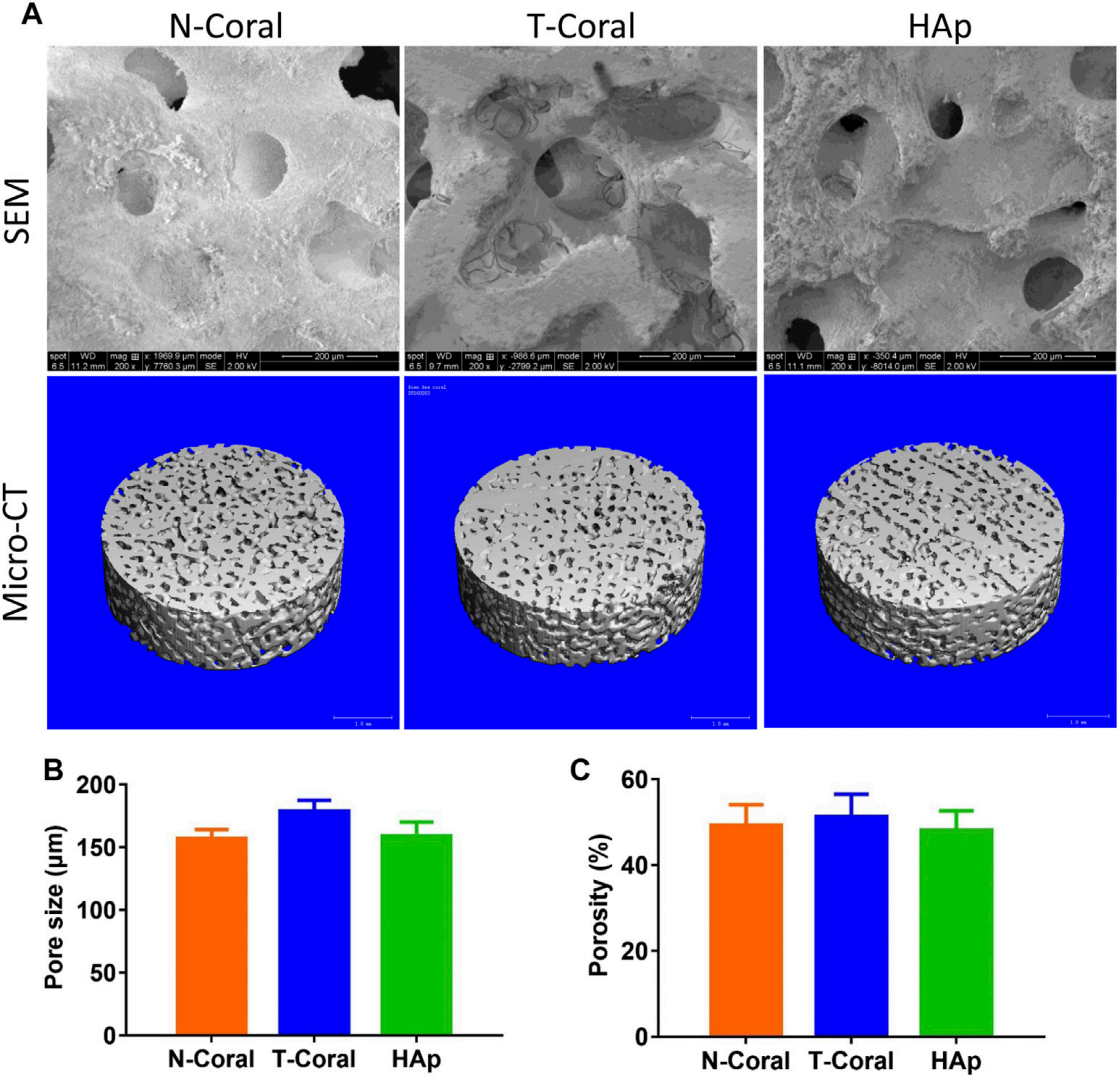
Surface morphology and microstructure of the porous scaffolds. **(A)** SEM images in 200-fold magnification or micro-CT 3D reconstructed images of the scaffolds. **(B)** Pore size as determined by SEM. **(C)** Porosity as determined by ethanol immersion method. Data were presented as mean ± SEM, *n* = 3.

As shown by the images of SEM and micro-CT, interconnected porous structure was found in all the scaffolds (Figure 3). The mean pore size was 157.0, 178.8 or 159.0 μm (Figure 3B) as determined by SEM in N-Coral, T-Coral, or HAp group, showing no significant difference among the groups. Regarding the surface morphology, N-Coral had many micro-dots on the surface, while T-Coral showed microcracked surface after hydrothermal treatment. As measured by the ethanol immersion method, the porosity was 49.28%, 51.35%, 48.12% (Figure 3C), showing no significant differences among the group.

### 3.3 Cell proliferation and differentiation

**FIGURE 4:**
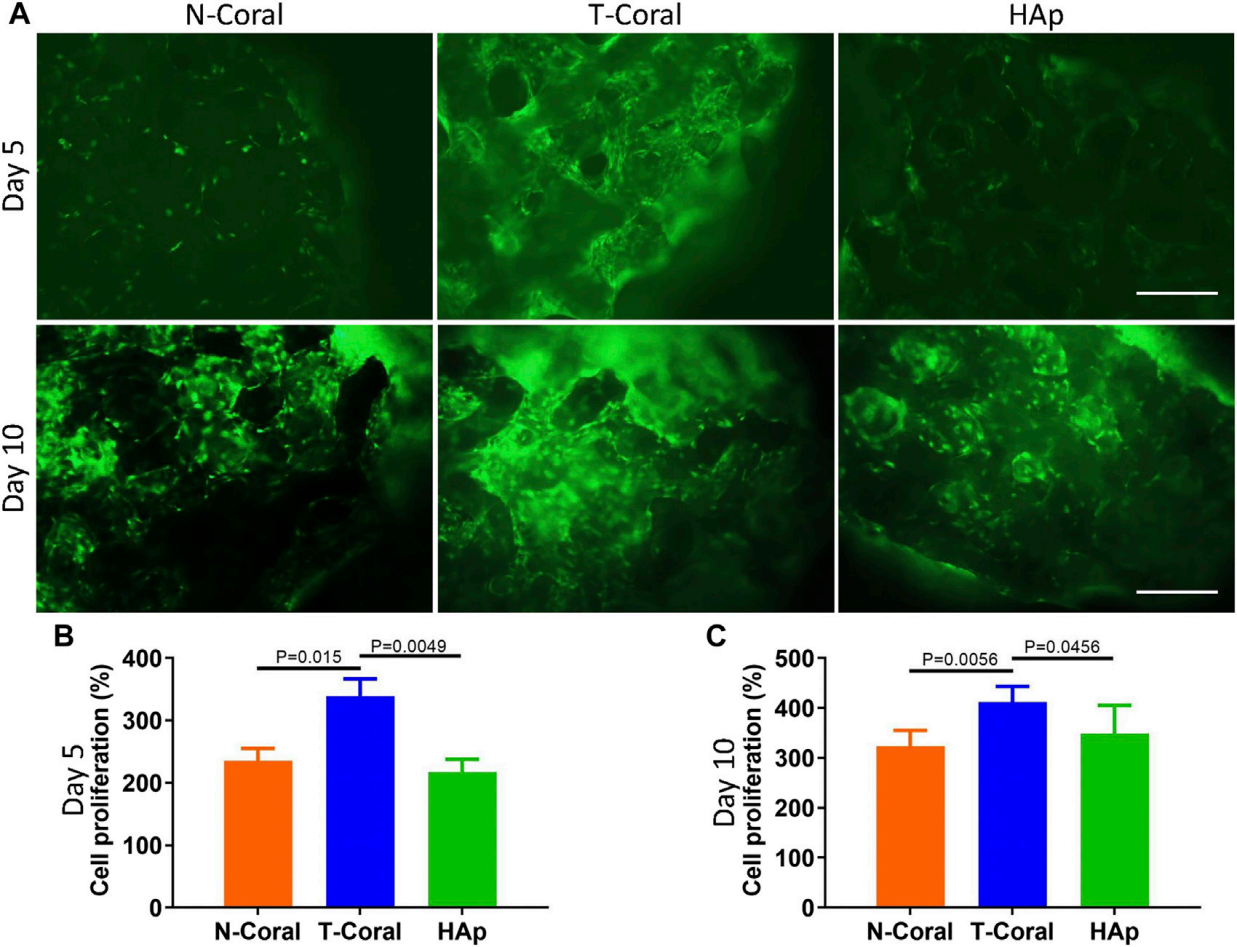
Cell proliferation on the surface of porous scaffolds. **(A)** Cellular fluorescent images of GFP expressing MSCs 5 days or 10 days after seeded on the porous scaffold and cell proliferation results as measured by Alamar Blue assay on day 5 **(B)** or day 10 **(C)** scale bar: 200 μm. Data were presented as mean ± SEM, *n* = 4.

**FIGURE 5:**
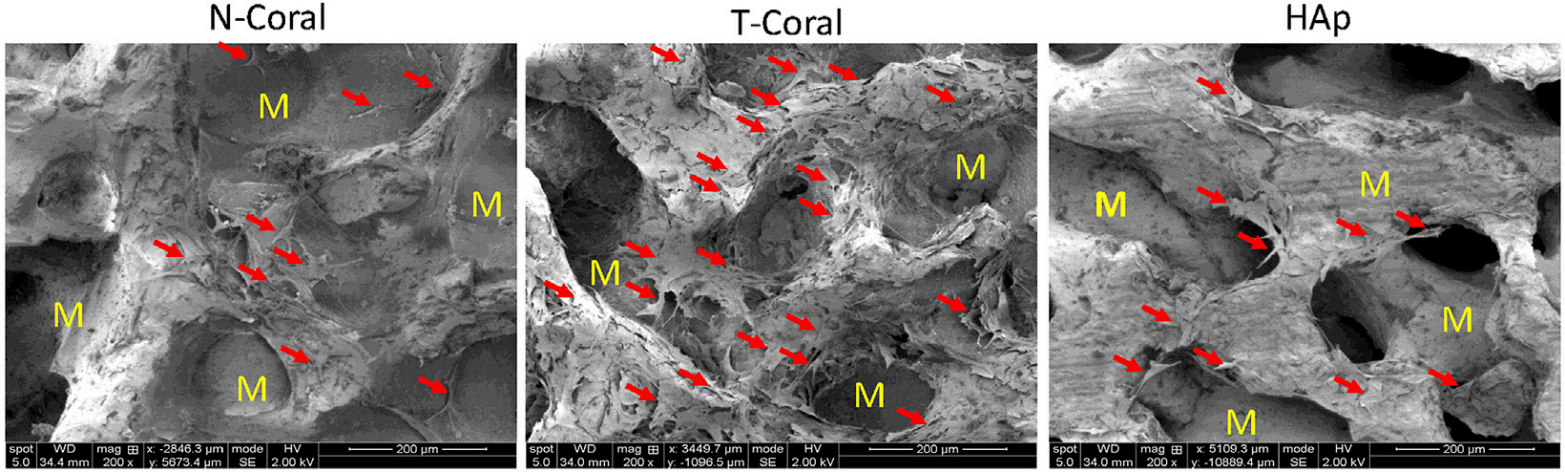
SEM images of GFP expressing MSCs seeded on the porous scaffolds on day 5. Red arrows indicate attached cells and M represents material. Magnification: ×200.

**FIGURE 6:**
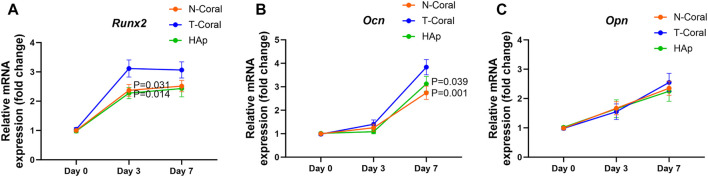
Gene expression of osteogenic markers of the MSCs seeded on the porous scaffolds. After confluent, the cells were induced with osteogenic medium for 0, 3 or 7 days. The RNA expressions of *Runx2* **(A)**, *Ocn* **(B)**, and *Opn* **(C)** were measured. Data were presented as mean ± SEM, *n* = 6.

Bone marrow derived MSCs expressing GFP were seeded onto the surface of the porous scaffolds, and the proliferation of MSCs were measured within 10 days (Figures 4, 5). Interestingly, as shown by the results of Alarma Blue assay, the cells proliferated much more vigorously in the T-Coral group, showing significantly higher cell proliferation rate than N-Coral (339.1% vs. 285.5%, *p* = 0.015) or HAp (339.1% vs. 217.5%, *p* = 0.0049) on day 5 (Figures 4A, B), and significantly higher cell proliferation rate than N-Coral (421.0% vs. 323.2%, *p* = 0.0056) or HAp (421.0% vs. 438.7%, *p* = 0.0456) on day 10 (Figures 4A, C) than the N-Coral or HAp group. The SEM images also confirmed that much more cells attached to the surface of T-Coral than other two groups (Figure 5). The gene expression results showed a significantly higher expression of *Runx2* (*p* = 0.031 & *p* = 0.014) after 3 days, and higher expression of *Ocn* (*p* = 0.001 and *p* = 0.039) after 7 days of osteogenic differentiation in the T-coral, comparing to the N-coral and HAp groups (Figure 6).

### 3.4 Bone repair in animal model

**FIGURE 7:**
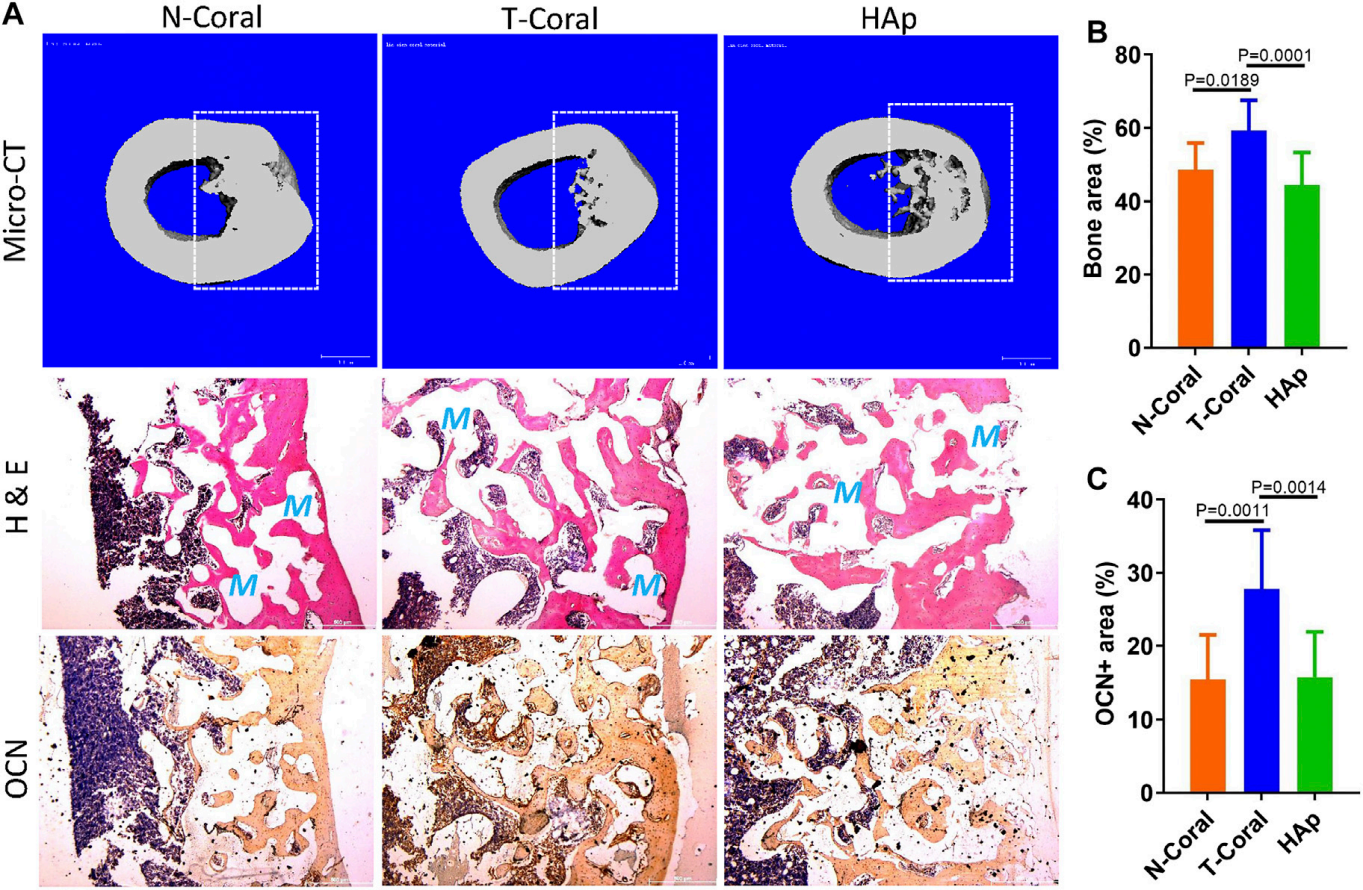
Repair effect of different porous scaffolds implanted in femoral shaft in rat for 8 weeks. **(A)** Representative cross-sectional images of micro-CT reconstruction (top), H & E staining (middle), and expression of osteocalcin (OCN, bottom). Areas within white dashed lines indicate the implant sites or region of interest for quantitative measurement. Red S indicate scaffold area after decalcification. **(B)** Semi-quantitative percentage of bone area measured by ImageJ from the histological results. **(C)** Semi-quantitative percentage of OCN positive area measured by ImageJ from the histological results. Scale bar: 500 μm. Data were presented as mean ± SEM, *n* = 10.

After 8-week of implantation, bone repair in the mid-shaft were determined by micro-CT analysis and histological examination. The cross-sectional 3D reconstructed image of the bone tissue demonstrated a continuous structure, with seamless integration between the implant and the bone tissue in all the groups (Figure 7A). Because of a confront effect of mineral composition in the scaffolds, quantitative micro-CT analysis was not sufficient to compare the differences in new forming bone among the groups. New bone formation was evidenced by H & E staining, showing much higher bone area in the T-Coral group comparing to the N-Coral (59.2% vs. 48.7%, *p* = 0.0189) or HAp group (59.2% vs. 44.8%, *p* = 0.0001) (Figures 7A, B). In addition, expression of bone formation marker osteocalcin (OCN) was significantly increased in the T-Coral group comparing to the N-Coral (27.8% vs. 15.4%, *p* = 0.0011) or HAp group (27.8% vs. 15.7%, *p* = 0.0014) (Figures 7A, C).

**FIGURE 8:**
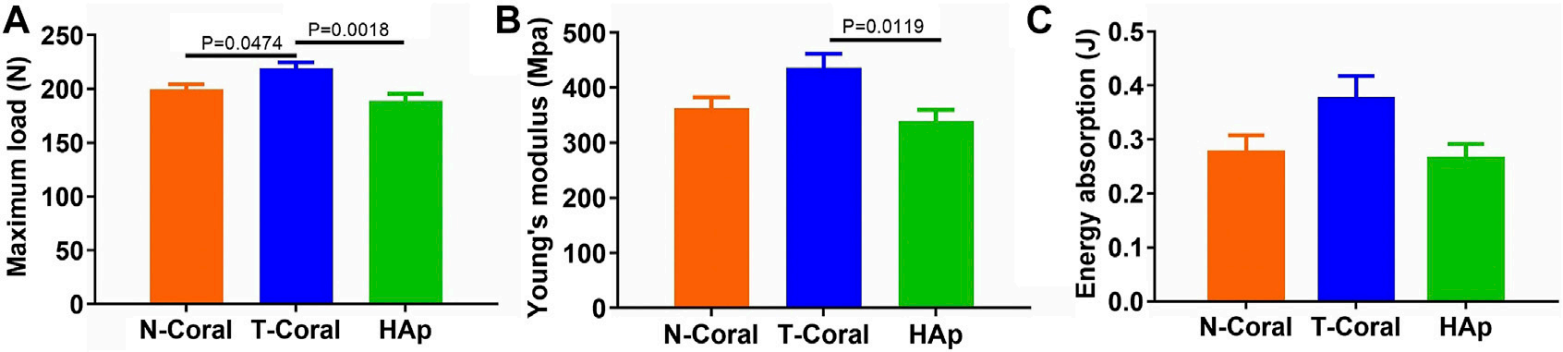
Results of bone biomechanics including maximum load **(A)**, Young’s modulus **(B)**, and energy absorption **(C)** in the affected femurs. Data were presented as mean ± SEM, *n* = 10.

More importantly, results from four-point bending mechanical test reveal that significantly higher maximum load in the T-Coral group than the N-Coral (219.2 vs. 199.7, *p* = 0.0478) or HAp group (219.2 vs. 189.1, *p* = 0.0018) (Figure 8A). And a significantly higher Young’s modulus could be found in the T-Coral group than the HAp group (436.1 vs. 338.9, *p* = 0.0119) (Figure 8B). However, there is no significant differences in the energy absorption among the groups (Figure 8C).

## 4 Discussion

In this study, we have synthesized a hydrothermally treated coral scaffolds (T-Coral), which showing comparative components as pure HAp, suggesting a successful conversion of nature coral to HAp after hydrothermal treatment. Moreover, the microporosity and interconnection were retained in the T-Coral. Interestingly, the T-Coral took advantages in cellular biocompatibility and bone repair in the bone defect model to HAp as well as N-Coral, indicating a potential alternative in the clinical application to pure HAp.

An ideal bone graft for bone tissue engineering should possess exceptional biocompatibility, which is considered a fundamental prerequisite. Recent effort has been made on improving the biocompatibility of bone grafts to enhance their effectiveness in tissue engineering and regenerative medicine application (Liu et al., 2022; Xu et al., 2022). Natural materials such as collagen, chitosan, and silk fibroin exhibit favorable biocompatibility by promoting cell adhesion, tissue regeneration, and integration within the body. Additionally, their biodegradability allows for tissue remodelling (Joyce et al., 2021). Besides of natural materials, surface medication techniques (Qiu et al., 2014) or nanotechnology-based approaches (Mohammadi et al., 2018) have also been adopted to improve biocompatibility of synthetic bone grafts by increasing the cell-graft interaction and tissue-graft integration. Coral is a ceramic form of natural matrix (Nandi et al., 2015), which may also provide biological cue for cell adhesion (Lalzawmliana et al., 2019). In this study, we found stem cells grew vigorously on the surface of N-Coral, T-Coral, and HAp. Superior cell proliferation was specially observed in the T-Coral group, suggesting a hydrothermal treatment may enhance the biocompatibility of the natural coral. One of the key changes that occur during hydrothermal treatment is the removal of organic components and impurities present in the coral scaffold. This process effectively eliminates potential sources of immunogenicity and cytotoxicity, reducing the risk of adverse reactions when the graft is implanted in the body.

Moreover, the hydrothermal treatment alters the surface topography of the coral graft, creating a roughened or porous structure. The positive effects of biomaterial surface roughness on cellular response, such as enhanced cell adhesion, proliferation, and differentiation, are widely recognized (Kazimierczak and Przekora, 2020; Stepanovska et al., 2020). To create porous and rough surfaces on bone implants, subtractive methods are commonly employed. Previous evidence showed that microcracked HAp specimens exhibit much more osteoblast attachment than non-cracked HAp (Shu et al., 2014), suggesting that microcracked surface on the T-Coral induced by hydrothermal modification may contribute to the enhanced cell attachment and proliferation. Here, we also observed a higher osteogenic differentiation potential of MSCs seeded on the surface of T-Coral comparing to the N-Coral or HAp. In addition, histological results from animal study showed a significantly higher bone area in bone defect sites. Previous study demonstrated that mineral deposition observed under confocal laser scanning microscope exhibited preferential mineralization at microcrack indentation sites of HAp (Shu et al., 2014). Another study showed that carbon fiber-reinforced polyetheretherketone-nanohydroxyapatite composite with optimal surface roughness favored osteogenesis *in vitro* and osseointegration *in vivo* (Deng et al., 2015). These results suggest that T-Coral may have a better osteoconductive properties than either N-Coral or pure HAp. However, previous study indicated that hydrothermal treatment can alter the Ca/P ratio, which may impact its biological performance (Onoda and Yamazaki, 2016). Further study is needed to evaluate the changes in Ca/P ratio, which is crucial for optimizing the material’s properties and suitability for biomedical applications.

As stated above, due to the many uncontrollable factors of natural coral materials, researchers currently study ways to improve the material’s composition by modifying the components or loading with cells or growth factors. Similar studies have also suggested that coral materials could be a potential bone grafts after modifications. An early report suggested that coral scaffold only could not repair cranial bone defect in a canine model, however, additional adipose derived stem cells could significantly increase the bone repair area (Cui et al., 2007). Ben-Nissan et al. reported a two-stage approach to attain nano-coated coralline HAp (Ben-Nissan et al., 2004). First, natural coral was completely converted to pure HA. Second, the micro- and nano-pores within the porous material were covered by a so-gel derived HAp, while large pores were maintained. Results from mechanical test showed biaxial strength was improved in the nano-coated scaffold. However, they did not-test any biological response. Roh et al. found that a mixture of silicon-substituted coral HAp with β-tricalcium phosphate in special ratios (60:40 or 50:50) significantly enhanced calvarial bone repair in a rat model (Roh et al., 2016). The silicon-substituted coral HAp was made from HAp converted from nature coral composed of 99% calcium carbonate, which further substituted by silicon. Recently, Decambron et al. reported the treatment of sheep bone defect model by applying coral granules with bone marrow derived MSCs or with additional BMP-2, and the results suggest that bone regeneration in critical-size bone defects could be achieved using coral-based tissue-engineered constructs (TECs) in the sheep model, however, nonunion still occurred in nearly half of the bone defects, suggesting further refinement of this therapeutic strategy is needed (Decambron et al., 2017a; Decambron et al., 2017b). Nandi et al. compared bone regeneration potency among hydrothermally converted coralline HAp scaffolds without growth factor, or with insulin like growth factor-1 (IGF-1) or bone morphogenetic protein-2 (BMP-2) (Nandi et al., 2015). The findings indicate that coralline scaffolds incorporating IGF-1 and BMP-2 facilitated the ingrowth of osseous tissue, initiation of bone healing and achieved complete integration between implants and natural bone.

The advantages of coral for cell growth and the extension of new blood vessels are due to its porous network structure, which is similar to that of bone tissue (Kang and Chang, 2018). This allows new bone to fuse tightly with the host bone tissue, facilitating the rapid restoration of normal bone tissue structure (Green et al., 2014). This study has shown that coral treated with hydrothermal modification maintains a porous structure with a uniform and interconnected pore distribution, similar to native bone. Although some microcracks were observed on the surface of the T-Coral, they did not compromise the overall structure of the coral material, suggesting that hydrothermal modification is a safe and effective approach. The clinical potential of hydrothermally treated coral scaffolds is extensive, including bone defect repair, oral implantology, and periodontal regeneration. The scaffold’s porous structure and bioactivity facilitate cell infiltration and bone tissue regeneration, promoting both bone defect healing and implant stability.

With approximately 70% of the Earth’s surface being covered by oceans and containing 90%–95% of the biosphere’s volume of living organisms, the ocean offers an extensive array of biological diversity and resources (Macha and Ben-Nissan, 2018). Despite its numerous advantages, coral stands out as a distinctive natural biological organism and plays a crucial role in sustaining marine ecosystems by providing habitats for various marine organisms (Keller et al., 2009). How to keep a balance between marine ecosystem and development of marine-based healthcare product becomes an important issue (Vicki, 2003; Rhyne et al., 2014). Sustainable collection practices help to minimize the impact on natural coral populations and maintain ecological balance in coral reef ecosystems.

## 5 Conclusion

In conclusion, this study suggests that hydrothermal modification enhances the cytocompatibility and therapeutic capacity of coral without altering its physical properties, showing superior effectiveness to synthetic HAp, highlighting the translational potential in promoting bone defect healing.

## Data Availability

The raw data supporting the conclusion of this article will be made available by the authors, without undue reservation.
